# Exploring the Dilemmas, Challenges, and Opportunities of Adolescent Fatherhood: An Exploratory Case Study

**DOI:** 10.1177/00469580221146827

**Published:** 2023-05-08

**Authors:** Eugene Musiiwa Makhavhu, Tendani Sara Ramukumba, Mmajapi Elizabeth Masala-Chokwe

**Affiliations:** 1 Nursing Science Department Sefako Makgatho Health Sciences University, Pretoria, South Africa; 2 Adelaide Tambo School of Nursing Sciences, Tshwane University of Technology, Pretoria, South Africa

**Keywords:** adolescent, fatherhood challenges, fatherhood dilemmas, teenage fatherhood, teenage pregnancy

## Abstract

Teenage pregnancy is a significant concern for society, and the effect on education is immense. In South Africa, policies were thus introduced allowing pregnant school-going children to continue attending class until the baby’s birth. However, research on adolescent pregnancy generally ignores teenage fathers and focuses mostly on teenage mothers. Parents of teenage girls are also encouraged to offer support to their children, but the same cannot be said about adolescent fathers. They confront numerous barriers in fulfilling their parenting roles. A qualitative exploratory study was conducted to explore adolescent fathers’ dilemmas, challenges, and opportunities. Interviews were conducted to collect data from 5 adolescent fathers in 1 township in South Africa. Findings indicate that adolescent fathers face various challenges and experience fatherhood differently. The phenomenon’s effects on education are immense and unavoidable, yet some opportunities accompany the fathering role. Adolescent fathers are exposed to several complex situations that impact their lives. To understand these, further research studies into adolescent fatherhood still need to be conducted, and reproductive health education efforts should equally be directed toward empowering boys to the same extent as girls.

**What is already known about this topic?**

Adolescent fatherhood is a topic not widely researched; in fact, when speaking of adolescent parenting, it is a norm that the topic mostly refers to teenage motherhood. As a result, adolescent fathers’ navigation through fatherhood is not widely covered.

**How does this research contribute to the field?**

This research offers insight into adolescent fatherhood in a low-resourced community from the fathers’ perspectives.

**What are your research’s implications toward theory, practice, or policy?**

This research can promote policy change, particularly in terms of the educational and social support adolescent fathers require.

## Introduction

Discussions on teenage parenting often focus on teenage mothers to the exclusion of teen fathers. There is also literature on teenage mothers’ influence on children’s outcomes, but the relationship between teenage fatherhood and children’s health and development is less documented.^1^ Moreover, although research is available about adolescent parenting, it generally ignores the father’s involvement and focuses more on the teenage mother. This may be attributed to data suggesting teenage girls are often impregnated by older men.^2^

Some negative societal perceptions regarding adolescent fathers reflect these young men are never ready to take on the parental role and are seldom involved in their children’s lives. These adolescents often deny paternity and remain absent from their children’s lives. However, it has also been reported that many adolescent fathers have a strong need to be active parents.^3^ Thus, while they are initially typically reluctant to come forward, some do so over time; others remain uninvolved and uninterested. Literature on adolescent fathers has highlighted negative life outcomes that may lead to adolescent fathers’ absenteeism from their children’s lives.

Some perceptions are that mothers’ presence influences the child’s development to a greater extent than the father’s. However, it has also been argued that a father or father figure’s presence in a child’s life positively affects the child’s life prospects, academic achievement, physical and emotional health, and linguistic, literary, and cognitive development.^4^ Moreover, adolescent fathers’ lives are often complicated, and they face many harsh realities.^5^ Some of these negative life outcomes include delinquency, lower levels of education, and reduced or lack of employment opportunities.^6^

It is well understood that adolescent parenting is not ideal as it is frequently characterized by negative outcomes.^7^ Researchers further report that adolescent fatherhood decreases years of schooling and the likelihood of receiving a high school diploma. Adolescent fathers often do not attend school, are unemployed, and seldom receive formal training compared to their childless peers.^8^ It is within this context that the investigation of dilemmas, challenges, and opportunities related to adolescent fatherhood (particularly in a low-resourced community) was pertinent.

## Methods

### Study Design and Setting

A qualitative exploratory design was employed in this study. Five participants aged between 18 and 25 who had fathered children before their 20th birthday were recruited from a mobile clinic operated by the university where the primary investigator was employed. The community did not have any other healthcare facilities within a 5-km radius.^9^ The clinic was funded by a philanthropic group and was the only source of healthcare in the community. The clinic has been operating since 2005 and offers primary healthcare services, including sexual health services and free condoms. A semi-structured interview,^10^ which was self-developed based on the available literature, was used to collect data. Participants were permanent residents of the low-resourced community of Soshanguve extensions 12 and 13 in the City of Tshwane, South Africa, where the mobile clinic was in operation. The area is in an extremely disadvantaged and recently formalized region with extensive informal settlements around its periphery.^11^

### Population and Sampling

Participants were sampled because they fit the study’s inclusion criteria. One participant who had attended the clinic for healthcare services was contacted to take part in the study and assisted the primary investigator in recruiting another participant known to him. This process resulted in snowball sampling,^12^ which ensued until the last participant was selected.

### Data Collection

Information leaflets were distributed to all participants to read and sign prior to data collection. Participants were prepared and informed of what the study entails and how interviews would be conducted. Data were collected by the primary investigator in English and translated to Setswana for participants who required such translation; Setswana is a dominant language in the community. Face-to-face semi-structured interviews were conducted and audio recorded with participants’ permission. Data were collected until data saturation was achieved^13-15^ and the research focus was met. It was challenging to sample adolescent fathers, as they are typically hard to identify, contact, and recruit to participate in research for several reasons.^16-18^

### Data Analysis

The primary investigator verbatim transcribed and analyzed the data using Tesch’s approach to content analysis. It is defined as a data analysis method that examines communication messages that are usually in written form.^19^ The main approach to data analysis involved a detailed exploration and review of each interview. Initial findings were coded to find the deeper meaning of the participant’s responses. Each new interview transcript was compared to the previous one to confirm or disapprove evidence, and common themes and subthemes were noted during the analysis. An independent coder also assisted to confirm or contradict the common emergent themes from analyzed data.

### Ethical Considerations

Ethical principles of nursing research were applied in the study. Informed consent was obtained from participants, and a copy of the information leaflet was shared with them for reference. Interviews were conducted in a private room provided by the mobile clinic to ensure privacy during data collection. Participants’ identities were protected, and recorded interviews were kept confidential. Anonymity was ensured by not identifying participants and using codes to report on the data. Information and arrangements contained in the participants’ information leaflet were adhered to and no changes or adaptations were made during the study period. Participants were assured of their right to self-determination and were further informed that interviews would be audio rerecorded.

## Results

### Socio-Demographic Information

**Table:** 

Participant	Age (years)	Age when the child was born (years)	Education	Employment status
1	18	17	Ninth grade: Continuing	Scholar
2	21	18	Ninth grade: Drop out	Unemployed
3	25	19	Further education and college training: Drop out	Permanently employed
4	25	19	Tenth grade: Drop out	Unemployed
5	24	18	Twelfth grade: Drop out	Unemployed

### Dilemmas of Fatherhood

Upon finding out about the pregnancy, the adolescent fathers’ reactions differed. Their dilemmas reflected the choices participants faced between undesirable alternatives, and included denial of the pregnancy and refusal of paternity.

#### Denial of pregnancy and refusal of paternity

Upon being informed of the pregnancy, some participants in this study denied the situation, although having engaged in sexual intercourse with the mother. They denied being responsible for the pregnancy when it was first announced. Participants were also unprepared to become fathers, as illustrated by their initial request to terminate the pregnancy.

P2: *It was tough. In the beginning I refused and told my partner that I am not the father of that child, and that maybe she was busy with other men. But you have to understand, though I knew the child was mine, I was just scared.*

P3: *I refused paternity and only accepted after seven months when I was calm. I think I was just scared and unsure.*

P4: *The first time she told me she was pregnant; we had a very serious and long fight and lots of disagreements. I was not ready then to have a child, I was pushing her to have an abortion and telling her that we are still very young to become parents and that we were not ready.*

#### Confusion and shock

Some participants indicated confusion after the pregnancy announcement as there was no expectation of a pregnancy at the time. They further mentioned feeling shocked at having had sex only once and were confounded about how that could lead to a pregnancy.

P1: *I was confused and uncertain. I did not know how I was going to tell my family and I was asking myself how they [the family] would respond to the pregnancy, especially because I am still at school but I had to accept the matter and took a decision to change*

P5: *I froze just after she said it, it’s not something I expected at that time. We had only done it [had sex] once, so I was a bit surprised that she could be pregnant already*

#### Failed relationships with the partners

The study’s findings further indicated that adolescent fathers separated from the mothers of their children after the baby’s birth. Participants highlighted no longer being involved with the mothers of their children; they said the only reason they remained in contact was due to co-parenting responsibilities. In some cases, this included participants financially supporting the child. One participant emphasized that although they had no relationship, he had to be courteous to the mother of his child so he could maintain access to the child. Another participant indicated that his partner and child had moved to another province. Explanations for the failed relationships were not shared.

P1: *We are no longer together; the only thing that brings us together now is the child we have. And because I love my child, I have to see her, but only through her mother.*

P3: *Even though we do not have a ‘romantic’ relationship with the mother, but because I am still supporting my child financially, I have to see her through her mother. That is the only way that we relate, as mother and father not boyfriend and girlfriend*

P4: *She and I are no longer together. We broke up about 3 years ago, but for the sake of the child, we still keep communication. If we don’t do that, then I might not be able to see my child*

P5: *It [the relationship] ended after the child was born. But we still maintain contact so I can see the child*

P2: *We are in a distant relationship because she stays in another province now. So I go see her and the child now and then, like end of the month, I went to see them. We have a civil relationship considering that she stays on the other side of the country and I am here. We try our best to keep it that way for the sake of the child. If I don’t, I will not be able to see my child*

### Challenges of Fatherhood

The study’s findings highlighted some challenges that adolescent fathers experienced. Some reported educational challenges, family challenges, financial challenges, and challenges in accessing their child.

#### Educational challenges

The difficulty of not successfully completing their schooling was a common theme that emerged from participants. The primary reason cited for their interrupted schooling was the pressure of feeling the need to support their child financially. This implies that the adolescent fathers’ educational prospects and ability to complete school are affected by their parental roles. The balance between schooling and being a parent is difficult for adolescent fathers to maintain. One participant indicated a loss of concentration in school contributed to his decision to discontinue schooling, with the aim of seeking employment.

P1: *Schooling and parenting becomes very challenging. I’m still at school. I am in the 9* ^th^ *grade now. It’s even harder now because at my age, I should have finished high school already.*

P2: *I was still at school at the time of the pregnancy, but I had to drop out from the 9^th^ grade when the child was born, I started looking at small businesses in order to be able to provide for my child. I knew I had a responsibility and I couldn’t put it on someone else.*

P3: *When my child was born, I had already passed my 12^th^ grade. I was in college about to complete and get my diploma. My father then said he will not be able to support both me and the child and that as a man I had to make a plan. I was then forced to drop out of college to go look for a job and be able to support my child.*

P4: *Having a child and being at school did eventually affect me because the mother of the baby was later unable to manage on her own, I then started losing concentration at school and I eventually dropped out to look for a job so I can help her.*

P5: *It is difficult to continue with school not knowing if your child is okay, and if she had something to eat or not. I had to leave school and look for odd jobs like doing people’s gardens in order to make a little money to send to my child. That makes any father feel like a man*

#### Effects on family relations

In addition to adolescent parenthood’s challenging impact on education, adolescent fathers also mentioned how the pregnancy announcement affected their family relations. Participants shared that family reactions toward the pregnancy varied from disappointment, punitive responses, acceptance of the pregnancy and, in some cases, families reacted as though they did not care.

P1: *I stay with my mother only. Her response was just OK. It was as though she did not care, but I could see the disappointment in her eyes when she looks at me*

P2: *Informing the family of what you had done is very challenging. My family was very disappointed then. However, in the end they accepted because there was not much they could do. The child was already there when they found out and we could not ‘return the child’*

P3: *I faced so much difficulty at home. Although my family eventually accepted that I had a child, they cut me off financially in order to support my child. Therefore, they could not help me complete college and at the same time support my child. My father said in his own words that he will not support me and my child at the same time*

P4: *You went and had this child, so it means you are now a man and have to take responsibility. Those are the words my father told me, so then I accepted the ‘punishment’ as it were.*

P5: *Telling my family about the pregnancy was difficult, but to my surprise they [the family] didn’t react very negatively, they just told me that now that things are the way they are [with the pregnancy], I needed to get a job so I can support my child and they would help me where I couldn’t reach and wherever they could.*

#### Financial challenges

The lack of parental and family support ultimately strained the adolescent fathers’ financial situation. They reported financial difficulty when their children were born. They were typically unable to financially provide for their children’s needs, as most participants were not formally employed.

Adolescent fathers’ lack of financial income may also influence their ability or inability to maintain access to the child. In certain cases, it is the adolescent mother’s family that prohibits the adolescent father from having access to the child.

P1: *Mostly, financial challenges, not being able to provide for my child since I am still in school. Otherwise everything was fine.*

P2: *Financial challenges are also there. If the child is sick at any time of the day, one needs to arrange transport to take him to the hospital and wake neighbours who have cars and pay them. If I don’t have money, then what happens to my child?*

P3: *Sometimes money talks. Not being able to support my child financially was my biggest challenge.*

P4: *I faced so many challenges, I saw after I left school that my priority is that I have a child. I was not getting anything [financial assistance] at home anymore; all they [were] offering me is food. Everything else is for the child. I then asked myself, who will buy me shoes, who will buy me clothes? I needed to make a plan to get money*

P5: *Although there were a lot of challenges at that time, but the most difficult part was financial problems. The child needs diapers, clothes, medications, and other things and if I cannot supply that then I feel I have failed as a father, but again how do you provide the things if you are not working*

#### Access to the child

Conflict with the mothers sometimes hindered adolescent fathers’ access to the child. Although the participants in this study had access, not all had unlimited access to their children. Some participants said that their access to their child was primarily impacted by their financial means. They consequently physically delivered money or other necessities for the child and were then in a position to see the child during these deliveries. For 1 participant, access was difficult as the mother and child had moved to a distant province, and it was problematic for him to travel since he was unemployed. However, he did manage to visit them at the end of each month.

P1: *I still see my child fulltime, anytime actually*

P3: *Yes, I do still see my child every now and then. I do everything for my child. From preschool fees, to her clothes, I am doing my bit as a father. Next year she will start school, I will still continue with that as my duty*

P4: *It is difficult sometimes to see the child. Since the relationship with the mother ended, I have to see my child through her and only if her family agrees for me to see the child you see.*

P2: *We are in a distant relationship because she stays in another province now. So I go see her and the child now and then, like end of the month, I went to see them.*

### Opportunities

Although challenging, adolescent fatherhood may also provide opportunities for these young men. In this study, adolescent fathers noted that their situation taught them how to adapt to the pressures of fatherhood.

#### Transition to fatherhood

The sudden transition from childhood to adulthood—and ultimately fatherhood—prepared them for sudden growth and responsibilities. There was an unexpected need to change after the birth of their children.

P1: *Fatherhood to me means changing from a stage of childhood to a stage of fatherhood so that I can be able to raise my child in a proper manner. It is difficult already, so one needs to grow.*

P2: *Now life is not as it was before the child was born. A lot has changed since then. Back then when you have some cents you would think of buying a nice pair of jeans or some nice T-shirts. Now, it is not like that anymore. When you have 10 Rands, you remember that you have a child. So responsibility is a lot on you to take care of the little one. Life is just not as free as it was before I had a child. So yeah, one has to grow in that manner.*

P3: *Fatherhood to me means being there for my child. Not necessarily by financial means. I’m willing to do anything and everything just to see my child survive.*

P4: *Being a father to me means being more involved in my child’s life. It means I have to be a more responsible person now. Be a good father to my child, a better one than my own father was to me and learning how to take care of myself as an adult [now] and learning to take care of others [My child].*

P5: *To me, being a father is more about availability and accessibility to my child. It is no longer about me alone anymore; it means I have to grow up now. I need to be strong and be there for my child. It means I have to be present in my child’s life and play a fatherhood role*

#### Growth and the meaning of fatherhood

Adolescent fathers defined fatherhood as central to their personal experiences and gave it meaning. There was also an opportunity to learn from their circumstances and teach other adolescent males who might potentially find themselves in a similar situation. There was an element of growth and a change in behavior that participants experienced, followed by emotions that come with parenting. Participants also learned that they could care for someone other than themselves. Being able to define fatherhood and give it meaning gave adolescent fathers an opportunity to learn and grow from what others may term “their mistakes.” For the 1 participant still in school, it further created an opportunity to focus more on his education.

P1: *Being a father at my age taught me a lot about caring for another person. I would say that my child has taught me to be softer than I was [laughing]. I am now much more serious at school than I ever was because my child depends on it*

P2: *Being able to love someone and learning how to take care of them and knowing you can do anything and everything to protect them. That is real love and that is what my child taught me*

P3: *You learn a lot from your mistakes. I cannot begin to explain just how much my situation has taught me but now I know I am an example to other young people and they can see that there is no need to rush in life. I hope my mistakes teaches someone something out there*

P5: *Having a child taught me a lot about responsibility, love, and caring. I am trying to be a much better person now so I can be a good role model for my child. I know now how it feels like to put someone first*

*There was a further unintended lesson on financial literacy. Participants learned to take care of the little money they received through odd jobs due to the sudden emergence of a new dependent.*

P4: *I had to grow up very quickly, and now I am also learning to use money wisely whenever I have done some work somewhere because my child comes first*

## Discussion

The study explored the dilemmas, challenges, and opportunities of fatherhood from the perspective of adolescent fathers in a low-resourced community. It was a common finding that adolescent fathers, upon discovering the pregnancy, did not immediately accept it. There was an instantaneous decision not to father the child by rejecting paternity. Research in South Africa also reported that teen fathers have difficulty accepting they were to be fathers, and the first reaction is often denial.^16^ This phenomenon is also common in other African countries, particularly among unmarried couples. Ultimately, the fact that women in consensual unions are not legally married may give the fathers room to deny their children.^20^ Reasons for denying the pregnancy were not further investigated, but another study suggested that the denial of paternity on the part of the father was a way to evade responsibility. It illustrated a lack of maturity, and also related to gender commitment and societal norms depicting expectations.^21^ In this case, denying paternity may be associated with the societal norms and expectations of fathers as providers.

The pregnancy announcement elicited different reactions from participants. As an unexpected change in their lives, at the time of hearing about the pregnancy for the first time, shock was expressed. This is further supported by data on unmarried fathers that found when pregnancy was unexpected, there was a typical reaction of surprise, anger, and confusion.^22^ Anxiety and stress were also common among adolescent fathers when they discovered they were to be parents.^2^ Adolescent fathers are likely to be scared and shocked due to being unprepared for the pregnancy.^23^ This unpreparedness can further be followed by the unavoidable task of having to inform their parents and other family members that they had fathered a child.

Adolescent fathers’ biggest fear was informing their parents about the pregnancy and fathering a child at that age. The fear, in some cases, was not of physical punishment but rather emotional conflicts between them and their families.^24^ This was particularly prominent among participants from a low-resourced community where poverty is rife; the announcement of a new member to the family means an extra person will need to be fed, and accepting that may be challenging. The lack of parental support was one of the challenges adolescent fathers experienced during their journey to fatherhood, and their fear of not receiving support prevailed.

For those parents who eventually accepted the pregnancy, there was a likelihood that their family could take over and support the child financially. However, financial support would typically come at the expense of the young men’s education.^24^ This study also found that families who chose to support their adolescent son’s child did so by no longer providing for the adolescent father’s basic needs. This is a punitive response that the family displays toward the adolescent father, perhaps to discourage these young men from repeating the same mistake.

The study’s findings also reflected that adolescent fathers separated from the mothers of their children once the child was born. The lack of preparedness to become fathers may be a contributory factor to the separation or the end of the relationship that this study established. However, the study’s focus was not on the reasons for separation, and it is unclear why the adolescent fathers separated from the mothers of their children after childbirth. Still, separation was almost imminent and is similarly reported in literature. For the adolescent fathers in this study, despite their separation from the child’s mother, this did not always translate into an absence from the child’s life. Participants maintained a courteous relationship with the mothers of their children primarily to maintain access to the child. This finding is corroborated in literature, which found that although adolescent fathers maintained a civil relationship for the sake of the child, they were no longer in relationships with the mothers of their children.^25^ This dilemma is faced by most adolescent fathers and may play a crucial role in their ultimate access to the child. Financial difficulties may be a potential reason for the separation, as other researchers found that the young fathers’ inability to financially provide hampered relationships in 56% of study samples.^25^ The inability to financially support the child may result in access to the child being denied.

Various factors may impact adolescent fathers’ access to the child after birth regardless of the status of the relationship between them and the mother. This may include the maternal family denying the adolescent father’s access to the child based on their perceived belief of his inability to be a financial provider, or as a result of the young man’s initial denial of paternity. A study on young fatherhood and child support found that active fatherhood can be encouraged or discouraged by the maternal family controlling access to the child.^26^ The mother’s side of the family would immediately assume kinship and guardianship of the child at the thought that the father may fail as a provider since the mother is a teenager herself. This indicates the amount of power the maternal family may possess in the relationship between father and child.

It was further reported that unemployment among adolescent fathers challenged the idea of their role as a provider.^26^ Societal norms relating to gender roles place child-support responsibilities on men as fathers; therefore, the expectation from the mother’s family can be seen as morally acceptable in society. Ultimately, access to the child is not always prohibited, although it is sometimes restricted.

Adolescent fathers also have certain opportunities as a result of being a father. Participants were positively involved with their children except those who desired to be involved but were prohibited by the child’s mother.^24^ However, such involvement also requires adolescent fathers to appreciate and accept their transition to fatherhood, which presents an opportunity for growth. The transition to parenthood can be a challenging time, in which both mothers and fathers experience an increased risk of distress and depression.^7^ However, the participants in this study indicated that their transition to fatherhood came with opportunities for growth and financial education.

Although the main focus of this study was not on gender roles and societal expectations of fatherhood, the adolescent fathers’ transition to adulthood was affected by cultural and societal norms. These transitions were highlighted when some adolescent fathers discontinued schooling in order to seek work and provide for their children.

Literature indicates that adolescent mothers are most affected when it comes to their education. However, this does not exclude adolescent fathers. Adolescent fathers are less likely to finish high school than their childless peers, and they frequently feel they have to get a job.^27^ Furthermore, adolescent fathers had significantly higher failure rates in completing secondary education.^23^

Due to the cessation of schooling, adolescent fathers find it more difficult to secure employment in the future than their childless peers who likely completed tertiary education and may have good employment prospects. Difficult financial situations made it very hard for participants to play an active role in their children’s lives. The desperation of being financially viable and the societal expectations and gender roles that state men are providers may place a severe strain on adolescent fathers and be a promoter of criminal behavior in a crime-ridden and competitive low-resourced community.

## Conclusion

This study followed 5 adolescent fathers in a low-resourced, recently formalized community in the City of Tshwane, South Africa. The objectives were to assess the dilemmas, challenges, and opportunities adolescent fathers faced from the beginning of the pregnancy to after the child was born. The study’s findings showed that adolescent fathers go through complex situations and the complexities have an impact on their lives.

Research into adolescent pregnancy and parenting focuses mostly on adolescent mothers and less on fathers. Further research studies into adolescent fatherhood need to be conducted, and reproductive health education efforts should equally be directed toward empowering boys as much as girls. More needs to be done to establish support structures for adolescent fathers to assist them in continuing their education.

## Limitations

- The study only focused on a small context of a low-resourced community; therefore, the results may not be transferrable to a different context. A broader study covering other parts of the townships may potentially yield different results.
- The sample size in this study may be considered a limitation due to the challenges experienced in sampling teenage fathers. Thus, the study was more dependent on data adequacy coupled with literature support, and the information collected from the participants was adequate to meet the study’s objectives.

## References

[1] Mollborn S Lovegrove PJ. How teenage fathers matter for children: evidence from the ECLS-B. J Fam Issues. 2011;32(1):3-30. doi: 10.1177/0192513X10370110 21927527 PMC3172315

[2] Matlakala FK Makhubele MW Mashilo MW. Challenges of teenage fatherhood in Vaalbank, Mpumalanga province. Gender Behav. 2018;16(3):12013-12020.

[3] Swartz S Bhana A Richter L Versfeld A. Promoting young fathers’ positive involvement in their children’s lives. HSRC Policy Brief, January. 2013. Accessed September 20, 2017. http://www.hsrc.ac.za/uploads/pageContent/3323/03%20Young%20Fathers.pdf

[4] Mavungu ME. Provider expectation and father involvement: learning from experiences of poor “absent fathers” in Gauteng, South Africa. Afr Sociol Rev. 2013;17(1):65-78.

[5] Kiselica MS Kiselica AM. The complicated worlds of adolescent fathers: implications for clinical practice, public policy, and research. Psychol Men Masc. 2014;15:260. doi:10.1037/a0037043

[6] Breslin M. Delinquency and young fatherhood share some, but not all risk factors. Fam Plann Perspect. 1998;30(3):152-155.

[7] Fletcher JM Wolfe BL. The effects of teenage fatherhood on young adult outcomes. Econ Inq. 2012;50(1):182-201. doi:10.1111/j.1465-7295.2011.00372.x 22329053

[8] Cundy J. Supporting young dads’ journey through fatherhood. Soc Policy Soc. 2016;15(1):141-153. doi:10.1017/S1474746415000524 26740799 PMC4697293

[9] Ibebuike JE Van Belkum C Maja TMM . The lived experiences and needs of children in child headed households in low resource poor community in Soshanguve, South Africa. J Good Governance Sustainable Dev Afr. 2014; 2(1):61-83

[10] Grove SK Gray J. Understanding Nursing Research; Building an Evidence-Based Practice. 8th ed. Elsevier; 2023.

[11] Nelson Mandela foundation. Township of Soshanguve, City of Tshwane. 2007. Accessed May 20, 2017. https://www.nelsonmandela.org/news/entry/soshanguve-community-conversation

[12] Grove SK Gray J. Understanding Nursing Research; Building an Evidence-Based Practice. 7th ed. Elsevier; 2019.

[13] Kerr C Nixon A Wild D. Assessing and demonstrating data saturation in qualitative inquiry supporting patient-reported outcomes research. Expert Rev Pharmacoecon Outcomes Res. 2014;10(3):269-281. 10.1586/erp.10.30 20545592

[14] Malterud K Siersman VD Guassora AD. Sample size in qualitative interview studies: guided by information power. Qual Health Res. 2016;26(13):1753-1760. doi:10.1177/1049732315617444 26613970

[15] Sebele-Mpofu FY. Saturation controversy in qualitative research: complexities and underlying assumptions. A literature review. Cogent Soc Sci. 2020;6(1):1838706. http://www.editorialmanager.com/cogentsocsci.doi:10.1080/23311886.2020.1838706

[16] Madiba S Nsiki C. Teen fathers’ perceptions and experiences of fatherhood: a qualitative exploration with in—school teen fathers in a rural district in South Africa. Curr Pediatr Res. 2017;21(3):501-506

[17] Chideya Y Williams F. Adolescent fathers: exploring their perceptions of their role as parent. Soc Work. 2014;49: 213.

[18] Lemay CA Cashman SB Elfenbein DS Felice ME. A qualitative study of the meaning of fatherhood among young urban fathers. Public Health Nurs. 2010;27:221-231. 20525094 10.1111/j.1525-1446.2010.00847.x

[19] Brink H Van der Walt C Van Rensburg G. Fundamentals of Research Methodology for Healthcare Professionals. 4th ed. Juta; 2018.

[20] Iyakaremye I Mukamana L Umutoni J. Paternity denial and consequences on children in patriarchal society: situation in consensual couples in Rwanda. Child Youth Serv Rev. 2020;118:105357. doi:10.1016/j.childyouth.2020.105357

[21] Pedersen W Blekesuane M. Sexual satisfaction in young adulthood. Cohabitation, committed dating or unattached life? Acta Sociol. 2003;46(3):179-193.

[22] Turney L. The denial of paternity: pregnancy as a risk to ‘pure relationships’. Sociology. 2011;45(6):1110-1125. doi:10.1177/0038038511416151

[23] Ngweso S Petersen RW Quinlivan JA. Birth experience of fathers in the setting of teenage pregnancy: are they prepared? World J Obstet Gynaecol. 2017;6(1):1-7. doi:10.5317/wjog.v6.i1.1

[24] Hendricks L Swartz S Bhana A. Why young men in South Africa plan to become teenage fathers: implications for the development of masculinities within contexts of poverty. J Psychol Afr. 2010;20(4):527-536. doi:10.1080/14330237.2010.10820411

[25] Langa M Smith N. Responsible teenage fatherhood in a South African historically disadvantaged community. J Psychol Afr. 2012;2(22):255-258. doi:10.1080/14330237.2012.10820526

[26] Selebano N Khunou G. Early-fatherhood in White City, Jabavu, Soweto: a time-bound, contextual construct. Open Fam Stud J. 2014;6(1):24-30.

[27] Runzel T. How are teenage fathers affected by pregnancy? 2006. Accessed April 4, 2017. http://howtoadult.com/teenage-fathers-affected-pregnancy-6789937.html

